# Prenatal Deltamethrin Exposure-Induced Cognitive Impairment in Offspring Is Ameliorated by Memantine Through NMDAR/BDNF Signaling in Hippocampus

**DOI:** 10.3389/fnins.2018.00615

**Published:** 2018-09-04

**Authors:** Chao Zhang, Qinghua Xu, Xia Xiao, Weihao Li, Qiang Kang, Xiong Zhang, Tinghua Wang, Yan Li

**Affiliations:** ^1^Department of Women and Child Health, School of Public Health, Kunming Medical University, Kunming, China; ^2^Department of Pediatrics, Weifang Yidu Central Hospital, Shandong, China; ^3^School of Stomatology, Kunming Medical University, Kunming, China; ^4^Department of Hepatobiliary Surgery, The Second Affiliated Hospital of Kunming Medical University, Kunming, China; ^5^Department of Experimental Zoology, Kunming Medical University, Kunming, China

**Keywords:** deltamethrin, *N*-methyl-D-aspartate receptor, cognitive impairment, BDNF, CREB, TrkB

## Abstract

**Background:** Pyrethroids have been widely used in residential and agricultural areas. However, little is known about the effects of prenatal exposure to deltamethrin on cognition in early development of offspring. In this study, the effects of prenatal exposure to deltamethrin on learning and memory abilities, *N*-methyl-D-aspartate receptor (NMDAR) subunits, brain derived neurotrophic factor (BDNF), Tyrosine kinase B (TrkB) receptor, and phosphorylated cAMP response element binding protein (pCREB) in the hippocampus of offspring rats were investigated.

**Experimental Approaches:** Groups each of six female SD rats, as F0-generation, were administered with deltamethrin (0, 0.54, 1.35, and 2.7, 9 mg/kg), or memantine (10 mg/kg), or co-administered with deltamethrin (9 mg/kg) and memantine (10 mg/kg) daily by gavage during pregnancy. The learning and memory ability was evaluated using Morris water maze (MWM) task on postnatal day 21. The expression of NMDAR (GluN1, GluN2A, and GluN2B), BDNF, pTrkB/TrkB, and pCREB/CREB in hippocampus were assessed with western blotting.

**Results:** Prenatal exposure to a relatively low dose of deltamethrin (2.7, 1.35, and 0.54 mg/kg) had no impact on learning and memory abilities or the expression of NMDAR, BDNF, pTrkB, and pCREB in the hippocampus of the exposed offspring. The group treated with 9 mg/kg deltamethrin showed impaired cognitive abilities and decreased expression levels of GluN1, GluN2A, GluN2B, BDNF, pCREB/CREB, and pTrkB/TrkB in the hippocampus. However, the declined cognitive ability were ameliorated by memantine treatment with increased GluN1, GluN2A, GluN2B, BDNF, pCREB/CREB, and pTrkB/TrkB expression in the hippocampus.

**Conclusion and Implications:** Prenatal exposure to a relatively high does of deltamethrin (9 mg/kg) alters cognition in offsprings and that this cognitive dysfunction can be ameliorated by memantine treatment. Moreover, NMDAR/BDNF signaling may be associated with the effects of prenatal exposure to deltamethrin on cognitive ability in offspring.

## Introduction

As a series of high effectiveness and low mammalian toxic insecticide, pyrethroids have been widely used in residential and agricultural areas (Tu et al., 2016). Pyrethroids themselves and their metabolites have been found to exist in our surroundings, such as in soil, residues of food, and breast milk (Du et al., 2010; Saillenfait et al., 2015). Pyrethroids that cause T (tremor) syndrome are classified as type I pyrethroids, and that induce CS (choreoathetosis and salivation) syndrome are known as type II pyrethroids (Saillenfait et al., 2015). Exposure during gestation has recently gained increasing attention because relatively lower levels of detoxifying enzymes induce high sensitivity to pyrethroid exposure during fetal and early postnatal development which is the “brain growth spurt” phase (Mense et al., 2006; Lee et al., 2015).

Type I pyrethroids induce an extended opening of the channels (mainly sodium channel and calcium channel), and type II pyrethroids cause an even longer prolongation than type I pyrethroids (Lee et al., 2015). Previous studies have indicated that the regulation of various neuroproteins, such as growth-associated protein 43 (GAP-43), calcium/calmodulin-dependent kinases II (CaMKII), postsynaptic density protein-95 (PSD95), glutamate receptor 1 (GluR1), synaptophysin and tau were involved in the process of impaired neurodevelopment induced by pyrethroids (Lee et al., 2015). Deltamethrin is a type II pyrethroid that induces strong calcium influx and glutamate release (Breckenridge et al., 2009). Acute clinical features of neurotoxicity such as motor coordination, sensory response, and cognition (Husain et al., 1996; Aziz et al., 2001; Wolansky and Harrill, 2008), caused by prethroid exposure, have been widely reported (Weiner et al., 2009). In addition, a combination of laboratory and epidemiological studies has recently demonstrated that the neurotoxicity of pyrethroids contributes to neurodegenerative diseases such as Alzheimer’s disease, Parkinson’s disease, and dementia, possibly because of oxidative stress induced by pyrethroids (Imanishi et al., 2013; Hansen et al., 2017). However, very few studies have investigated cognitive dysfunction in offspring prenatally exposed to deltamethrin.

*N*-methyl-D-aspartate receptors (NMDARs), as excitatory glutamate receptors, possess a Ca^2+^ channel (Morikawa and Furuhama, 2009). NMDARs play a critical role in neuronal differentiation, memory formation, and learning. Previous studies have suggested a relation between decreased expressions of NMDAR subunits and impairment of learning and memory abilities (Babot et al., 2007). Moreover, NMDA receptor antagonists, such as MK-801, have neuroprotective properties in pyrethroid-treated neurons, which suggests the involvement of NMDARs in the mechanism of pyrethroid-induced neurotoxicity (Chugh et al., 1992; Umeda et al., 2016). Memantine, which is a non-selective, non-competitive antagonist of NMDA receptors, has been proved to improve cognitive abilities in several conditions, such as Parkinson’ disease, ischemia dementia, through regulating NMDA glutamate receptors (Olivares et al., 2012; Wang et al., 2016).

Brain derived neurotrophic factor (BDNF) plays an important role in neurodevelopment, synapse regulation, learning, and memory through the activation of NMDAR in hippocampus (Guo et al., 2015). The cAMP response element binding protein (CREB) signal pathway is well known to play a critical role in memory formation and participate in the BDNF pathway which elucidates cognitive function regulations in the hippocampus (Platenik et al., 2000). Repression of BDNF mRNA expression and Ca^2+^ influx due to pyrethroid exposure has been reported by Imamura et al. (2000).

Taking into account the possible neurotoxic effects of pyrethroids, this study investigated the potential cognitive impairment in offspring prenatally exposed to deltamethrin. In the present study, pregnant SD rats were administered daily with different doses of deltamethrin, co-administration of deltamethrin and memantine, or memantine alone. On postnatal day 21 (PND 21), Morris water maze (MWM) were utilized to observe the cognitive abilities in the offspring. The protein expressions of GluN1, GluN2A, GluN2B, BDNF, phosphorylated tyrosine kinase B (pTrkB) receptor, and phosphorylated CREB (pCREB), in the hippocampus were quantified by western blotting analysis. The results of the present study suggested that a high dose of deltamethrin (9 mg/kg) exposure during pregnancy resulted in cognitive impairment and altered expression of GluN1, GluN2A, GluN2B, BDNF, pCREB, and pTrkB in offspring on PND 21. The observed cognitive dysfunction was ameliorated by memantine treatment.

## Materials and Methods

### Animals

Male and female Sprague-Dawley (SD) rats weighing 220–250 g (7–9 weeks of age) obtained from Kunming laboratory animal center (Kunming, China) were used in this investigation. The animals were housed in plastic cases in a ventilated room with a controlled environment (22 ± 2°C, 55 ± 10% humidity, and 12-h light/dark cycle). The rats were provided with free access to food and water throughout the study. Animal handling and procedures followed the guideline of Chinese Academy of Sciences. As F0-generation, 42 female rats were evenly divided into seven groups: a control group, groups treated daily with deltamethrin (≥98% pure; Sigma-Aldrich, St. Louis, MO, United States) at a dose of 0.54 mg/kg, 1.35 mg/kg, 2.7 mg/kg, or a 9 mg/kg bw (equivalent to 1/250, 1/100, 1/50, 1/15 of the LD_50)_, a group treated with deltamethrin (9 mg/kg) mixed with memantine (10 mg/kg; H. Lundbeck A/S, Valby, Denmark), and a group treated with memantine alone. Female rats were mated overnight at the proportion of two females to one male. Pregnant rats were administered with different those compounds dissolved in coin oil (Yuanye, Shanghai, China) by intragastric administration daily beginning on the first day of pregnancy (vaginal smear positive) throughout the gestation period. Control animals were given the vehicle alone (coin oil) in the same way as deltamethrin-treated animals. Maternal weight was monitored daily during pregnancy. Pregnant rats were observed after deltamethrin treatment for clinical signs of pyrethroid toxicity such as seizures, salivation, and tremors. All pregnant rats were allowed to deliver and nurture their offsprings normally. The day of birth was determined to be postnatal day 0 (PND 0). Pups were weighed at PND 0, PND 7, PND 14, and PND 21. All six litters were used in each group. Variables such as litter size and the number of alive pups were assessed. At weaning (PND 21), male and female pups were housed separately. To control specific litter effects, four animals (half male and half female) were randomly selected from each litter for following tests.

### Morris Water Maze (MWM)

Twelve pups of each group (one male and one female from each litter) were selected randomly at PND 21, and MWM test was used to evaluate spatial discrimination and memory abilities in offspring through multiple attempts in searching a fixed hidden platform in the pool (90 cm of diameter), to which the water mixed with ink at 25°C was added. The pool was divided into four quadrants and the platform was put into the middle of the quadrant III. In place navigation test, rats were put into the water in a certain order. Duration from dropping into the water to reaching the hidden platform was observed and recorded as escape latency. If the rats could not find the platform within 120 s, it was recorded as 120 s. The test was conducted for five consecutive days. On day 6, the place probe trial was performed to determine the familiarity of the rats with the platform in the water maze. Each rat was subjected to a special probe trial (60 s) initially after removing the platform. Spatial memory ability was evaluated by the percentage of time that rats spent in the target zone (quadrant III) within 60 s. Data were recorded on computer with a video tracking system and analyzed with the SMART 3.0 software (Panlab/Harvard Apparatus, Barcelona, Spain).

### Western Blot Analysis

At 21 days of age, another set of 12 pups was randomly selected from each group (one male and one female from each litter). One side of the brain was used for western blot and the other side of brain was used for immunofluorescence assay. To examine protein expression levels of GluN1, GluN2A, GluN2B, BDNF, pTrkB, and pCREB, hippocampal tissues were homogenized in RIPA (Beyotime, Shanghai, China), and protein concentrations were measured using BCA assay kit (Beyotime, Shanghai, China). Protein (20 μg) was separated by SDS–PAGE at 80 V for 2 h, and then transferred onto PVDF membranes (Millipore, Billerica, MA, United States). The membranes were then blocked in 5% non-fat milk for 1 h at room temperature. Then, blocked membranes were incubated with primary antibody anti-GluN1 (dilution, 1:1,000; Abcam, Cambridge, MA, United States), anti-GluN2A (dilution, 1:1,000; Abcam, Cambridge, MA, United States), anti-GluN2B (dilution, 1:1,000; Abcam, Cambridge, MA, United States), anti-BDNF (dilution, 1:1,000; Abcam, Cambridge, MA, United States), anti-CREB (dilution, 1:1,000; Abcam, Cambridge, MA, United States), anti-phosphorylated (p)-CREB (dilution, 1:10,000; Abcam, Cambridge, MA, United States), anti-TrkB (dilution, 1:500; Abcam, Cambridge, MA, United States), anti-phosphorylated (p)-TrkB (dilution, 1:1,000; Abcam, Cambridge, MA, United States), and anti-β-actin (dilution, 1:1,000; Abcam, Cambridge, MA, United States) primary antibodies overnight at 4°C, followed by incubation with the secondary antibodies (dilution, 1:5,000; goat anti-rabbit IgG; ZSGB-BIO, Beijing, China) for 1 h at room temperature. The membranes were developed with enhanced chemiluminescence (ECL) kit and pictures were captured in a Bio-Gel Imagining System (Bio-Rad, Hercules, CA, United States). Protein levels in the different groups were presented as percentages of the control group.

### Immunofluorescence

For immunofluorescence assay, brains tissues from each group were harvested and then immersed in 4% formaldehyde solution for 24 h followed by gradient of dehydration in 10, 20, and 30% of sugar solution. The brain tissues were OCT-embedded and sliced into coronal sections by a frozen microtome (Leica CM1900, Wetzlar, Hesse-Darmstadt, Germany) at a thickness of 10 μm through the dorsal hippocampus. After the sections were permeabilized with PBS/0.1% Triton × 100, the sections were incubated in the mixture anti-NeuN (mouse, Abcam, 1:1000) and anti-BDNF (rabbit, Abcam, 1:750) at 4°C overnight, and then followed by fluorescence secondary antibody, including DyLight 488 affiniPure goat anti-mouse IgG (green) and DyLight 594 affiniPure goat anti-rabbit IgG (red). Then, the sections were washed with PBS and mounted by DAPI. Finally, the pictures were captured by a Nikon A1 confocal laser scanning microscope system (Nikon Corp., Tokyo, Japan).

### Statistical Analysis

All data were presented as means ± standard deviation (SD) and analyzed with SPSS 17.0. To assess statistical differences among groups in escape latency and swimming distance, data were analyzed by two-way repeated measures ANOVA (day × group). One-way ANOVA was utilized to analyze data obtained from multiple groups. Least significant difference (LSD) *post hoc* tests were used to identify differences between specific groups. *P*-value < 0.05 was considered statistically significant.

## Results

### Clinical Observations and Reproductive Outcome Data

Deltamethrin and/or memantine did not exhibit any sign of maternal toxicity. There were no death of female rats in any of the treated groups. And the female rats treated with deltamethrin did not show any clinical signs of toxicity such as seizures, salivation, and tremors. The maternal weight gain was similar in all groups (**Table 1**). The body weights of the pups in 9 mg/kg deltamethrin treated group were significantly higher on PND 0 [*F*(6,387) = 6.897, *P* < 0.01] than control group, but were unaffected on PND 7, PND 14, and PND 21 (**Table 1**). The number of alive pups and litter size were also unaffected (**Table 1**).

**Table 1:** Weight gain of dams and parameters of rat pups following prenatal exposure to deltamethrin and/or memantine.

	Control	9 mg/kg DM	2.7 mg/kg DM	1.35 mg/kg DM	0.54 mg/kg DM	9 mg/kg DM +MEM	MEM
**Maternal weight gain during pregnancy (g)**							
Days 0–7	14.1 ± 3.08	13.71 ± 2.95	13.31 ± 2.79	13.95 ± 2.35	13.71 ± 2.97	13.32 ± 2.42	13.51 ± 2.42
Days 7–14	14.76 ± 5.54	13.84 ± 5.51	13.45 ± 4.75	14.53 ± 5.26	13.01 ± 4.48	14.40 ± 4.47	14.26 ± 5.33
Days 14–21	65.39 ± 15.2	63.68 ± 15.59	64.09 ± 14.76	64.33 ± 13.8	65.55 ± 16.48	65.68 ± 13.27	66.7 ± 16.48
Overall weight gain during pregnancy (%)	40.31 ± 6.17	39.08 ± 6.18	38.77 ± 6.03	39.76 ± 5.49	39.36 ± 4.88	39.96 ± 4.4	40.32 ± 5.98
Number of litters	6	6	6	6	6	6	6
Litter size	10.0 ± 1.41	8.5 ± 1.38	9.2 ± 1.47	9.7 ± 1.97	9.5 ± 1.87	9.0 ± 1.41	9.8 ± 1.17
Live birth index (%)^a^	98.3	98	100	98.3	96.5	98.1	100
Weaning index (%)^b^	96.7	98	96.4	98.2	100	100	96.7
**Body weight of pups (g)**					
PND 0	5.67 ± 0.41	5.98 ± 0.38^∗∗^	5.78 ± 0.43	5.61 ± 0.43	5.53 ± 0.45	5.54 ± 0.48	5.72 ± 0.41
PND 7	13.53 ± 0.78	13.74 ± 0.75	13.65 ± 0.71	13.32 ± 0.75	13.53 ± 0.67	13.57 ± 0.74	13.50 ± 0.77
PND 14	27.62 ± 1.37	28.12 ± 1.50	27.78 ± 1.49	27.48 ± 1.38	27.32 ± 1.37	27.38 ± 1.19	28.09 ± 1.62
PND 21	57.10 ± 4.21	52.67 ± 4.72	49.73 ± 4.87	50.48 ± 4.40	49.46 ± 4.91	51.24 ± 4.48	52.00 ± 4.39

*Values are represented as means ± SD. ^*a*^Live birth index = (number of live pups on PND 0/number of pups delivered) × 100. ^*b*^Weaning index = (number of live pups on PND 21/number of pups alive on PND 0) × 100. ^∗∗^Significantly different from control group, *P*-value < 0.01*.

### Learning and Memory Ability Test by Morris Water Maze

**FIGURE 1:**
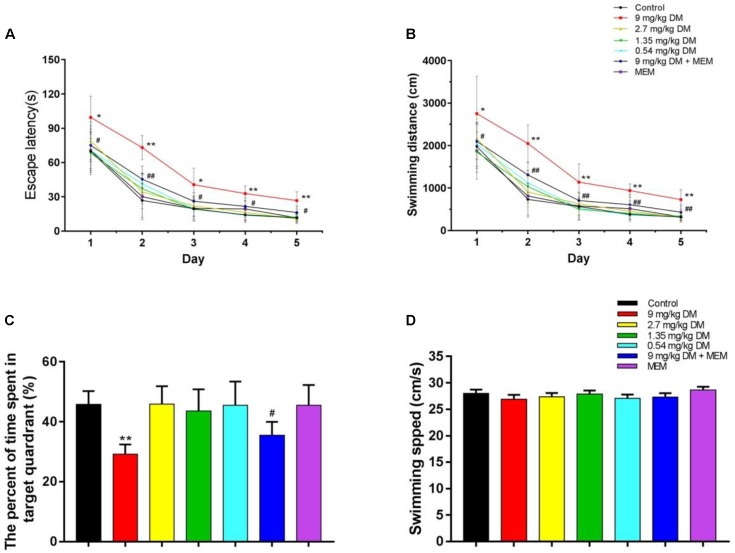
The evaluation of learning and memory ability in MWM test. The results of MWM test are shown: **(A)** escape latency, **(B)** swimming distance, **(C)** the percent of time spent in target quadrant, and **(D)** swimming speed. The group treated with the highest dose of deltamethrin (9 mg/kg) demonstrated significant learning and memory impairment compared with the control group, while the other deltamethrin treated groups (2.7, 1.35, and 0.54 mg/kg) did not. And the co-administrated of deltamethrin and memantine group ameliorated the cognitive impairment observed in the 9 mg/kg treated group. No significant differences in swimming speed were found between these groups. ^∗^*P* < 0.05 compared to the control group. ^∗∗^*P* < 0.01 compared to the control group. ^##^*P* < 0.01 compared to the 9 mg/kg DM group. ^#^*P* < 0.05 compared to the 9 mg/kg DM group. *n* = 12 in each group. DM: deltamethrin, MEM: memantine.

The present study examined whether prenatal exposure to deltamethrin impairs cognitive abilities in the offsprings. Two-way repeated ANOVA analyses of the data from escape latency revealed that there were statistically significant differences for day [*F*(4,308) = 401.523, *P* < 0.01], group [*F*(6,77) = 38.175, *P* < 0.01], and day × group interaction [*F*(24,308) = 1.939, *P* < 0.5]. In *post hoc* analysis, the escape latency in 9 mg/kg deltamethrin treated group was significantly higher than control group (*P* < 0.01). The escape latency of the group given both deltamethrin (9 mg/kg) and memantine (10 mg/kg) was lower than that of the group treated with 9 mg/kg deltamethrin alone (*P* < 0.01) (**Figure 1A**).

Data from swimming distance indicated that there were significant differences for day [*F*(4,308) = 347.31, *P* < 0.01], group [*F*(6,77) = 28.948, *P* < 0.01], and day × group interaction [*F*(24,308) = 1.901, *P* < 0.5]. LSD *post hoc* test exhibited that 9 mg/kg deltamethrin group showed longer swimming distance than control group (*P* < 0.01). And the mixed deltamethrin (9 mg/kg) and memantine (10 mg/kg) treated group showed shorter swimming distance than 9 mg/kg deltamethrin treated group (*P* < 0.01) (**Figure 1B**).

The spatial probe task was performed on day 6. The percentage of time spent in the target quadrant differed significantly between the seven groups [*F*(6,77) = 17.265, *P* < 0.01]. In the *post hoc* analysis, the percentage of time spent in the target quadrant in the 9 mg/kg deltamethrin administered group was significantly lower than that in the control group (*P* < 0.05). However, the group coadministered deltamethrin and memantine exhibited a significantly greater percentage of time spent in the target quadrant the group exposed to 9 mg/kg deltamethrin alone (*P* < 0.05) (**Figure 1C**). No significant differences were found in swimming speed between the groups [*F*(6,77) = 0.715, *P* > 0.05] (**Figure 1D**).

### Protein Expressions in Hippocampus Detected by Western Blot

**FIGURE 2:**
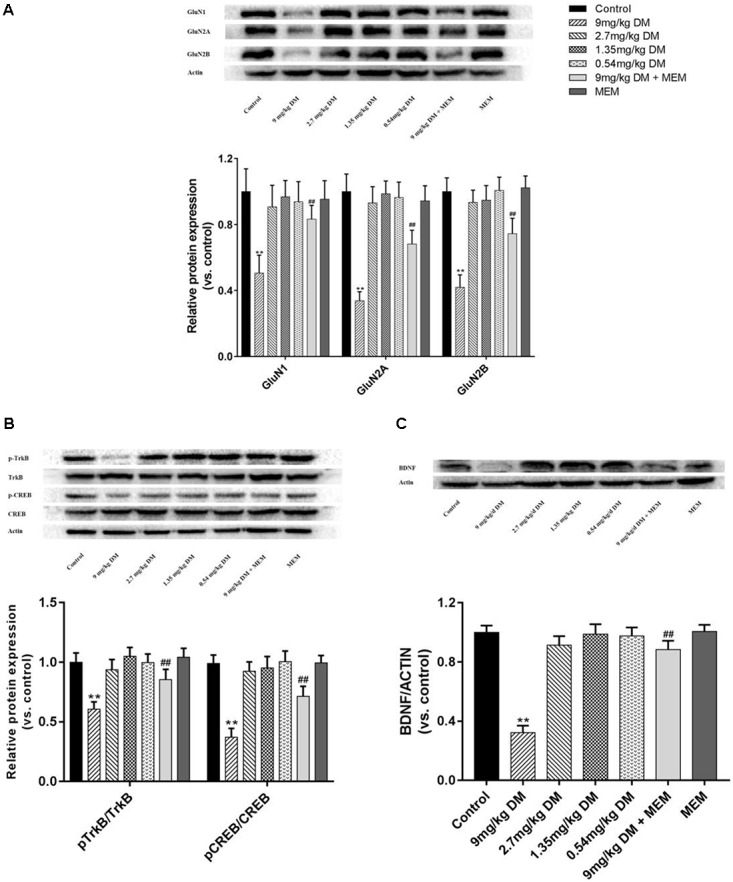
Western blot of protein expression of hippocampus GluN1, GluN2A, GluN2B **(A)**, pTrkB, TrkB, pCREB, CREB **(B)**, and BDNF **(C)** in the hippocampus of offspring. **(A)** Histogram showing the protein expression of GluN1, GluN2A, and GluN2B in the hippocampus (from left to right). Lanes (from left to right) represent the control, 9mg/kg DM, 2.7mg/kg DM, 1.35mg/kg DM, 0.54mg/kg DM, 9mg/kg DM + MEM, MEM groups. β-actin was used as control. **(B)** Western blot showing the protein expression of pTrkB, TrkB, pCREB, CREB and the column chart showing the quantification of the pTrkB/TrkB and pCREB/CREB ratio. **(C)** Western blot results of protein expression of BDNF in hippocampus of the offspring. β-actin was used as control. *n* = 12 in each group. ^∗∗^*P* < 0.01 when compared with control group. ^##^*P* < 0.01 when compared with 9 mg/kg DM group. DM: deltamethrin, MEM: memantine.

Oral administration of different doses (9, 2.7, 1.35, and 0.54 mg/kg) of deltamethrin to pregnant rats produced a dose-dependent changes in GluN1 [*F*(6,77) = 26.49, *P* < 0.01], GluN2A [*F*(6,77) = 92.28, *P* < 0.01], GluN2B [*F*(6,77) = 86.861, *P* < 0.01], pTrkB/TrkB [*F*(6,77) = 49.987, *P* < 0.01], pCREB/CREB [*F*(6,77) = 104.313, *P* < 0.01], and BDNF [*F*(6,77) = 244.754, *P* < 0.01] in the hippocampus of offspring postnatally at 21 day (**Figure 2A**). In the *post hoc* test, significant decreases in GluN1 (*P* < 0.01), GluN2A (*P* < 0.01), GluN2B (*P* < 0.01), BDNF (*P* < 0.01), pCREB/CREB (*P* < 0.01), and pTrkB/TrkB (*P* < 0.01) were observed in the hippocampus isolated from PND21 offspring prenatally exposed to a relatively high doses of deltamethrin (9 mg/kg), compared with the control group (**Figure 2B**). However, in the mixed feeding (9 mg/kg DM + MEM) group, significant increases of GluN1 (*P* < 0.01), GluN2A (*P* < 0.01), GluN2B (*P* < 0.01), BDNF (*P* < 0.01), pTrkB/TrkB (*P* < 0.01), and pCREB/CREB (*P* < 0.01) were observed compared with that in the 9 mg/kg deltamethrin group (*P* < 0.01; **Figure 2**).

### Immunofluorescence of BDNF

**FIGURE 3:**
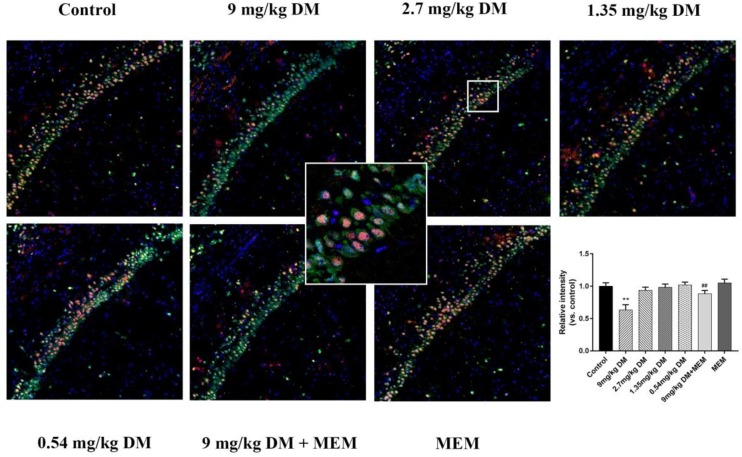
Immunofluorescence analysis of BDNF expression in hippocampus. Representative confocal micrographs of BDNF expression in the CA1 region (BDNF staining shown in red, neurons shown in green, and DAPI counterstain in blue). Immunofluorescence staining for protein expression of BDNF in the CA1 area of hippocampus in different groups: control group, 9 mg/kg deltamethrin treated group, 2.7 mg/kg deltamethrin treated group, 1.35 mg/kg deltamethrin treated group, 0.54 mg/kg deltamethrin treated group, co-administered of 9 mg/kg deltamethrin and memantien group, memantine group. Scale bar = 200 μm. Data are mean ± SD. *n* = 12 rats/group. ^∗∗^*P* < 0.01 compared to control group, ^##^*P* < 0.01 compared to 9 mg/kg deltamethrin treated group. DM: deltamethrin, MEM: memantine.

The expression of BDNF in neurons of CA1 region is shown in **Figure 3**. The *post hoc* analysis revealed that the expression of BDNF in 9 mg/kg deltamethrin group was significantly lower than in the control group (*P* < 0.01). No difference in BDNF expression was observed between the control group and the groups treated with the relatively lower doses of deltamethrin (0.54, 1.35, and 2.7 mg/kg) (*P* > 0.05). However, in the mixed treated group (9 mg/kg deltamethrin and 10 mg/kg memantine), BDNF expression was higher than that in the 9 mg/kg deltamethrin treated group (*P* < 0.01).

## Discussion

In the present study, we investigate the effects of prenatal exposure to different doses of deltamethrin on cognitive abilities in F1-rats and the expression of NMDARs, BDNF, pCREB/CREB, and pTrkB/TrkB in hippocampus. Consistent with previous studies, we found that deltamethrin has a dose-dependent effect on cognitive functions of F1-rats born to deltamethrin-treated rats (Aziz et al., 2001; Breckenridge et al., 2009). The offspring of rats exposed to the highest dose of deltamethrin (9 mg/kg) tested exhibited impaired learning and memory abilities in MWM tasks. In addition, exposure to the highest dose of deltamethrin (9 mg/kg) decreased the levels of GluN1, GluN2A, GluN2B, BDNF, pTrkB/TrkB, and pCREB/CREB. However, the declined cognitive ability were ameliorated by memantine treatment with increased GluN1, GluN2A, GluN2B, BDNF, pTrkB/TrkB, and pCREB/CREB expression in hippocampus.

In this study, maternal weight gain was not affected by deltamethrin and/or memantine treatment, and no obvious adverse effects on pregnant rats were observed. These results are in good agreement with previous studies suggesting absence of maternal toxicity induced by deltamethrin exposure (Lazarini et al., 2001; Andrade et al., 2002). In addition, the body weights of the pups at PND 21 were also unaffected between the different groups. Thus we suggested that deltamethrin and memantine had no behavioral and physiological effects on the dams.

Morris water maze is a reliable test to evaluate cognitive functions in rodent animals and can objectively assess changes in spatial learning and memory abilities in offspring. Previous studies have suggested that prenatal exposure to deltamethrin induced locomotor effects in offspring (Kung et al., 2015). Deltamethrin exposure has been reported to increase locomotor activity in adult mice and larval zebrafish (Eriksson and Fredriksson, 1991; Kung et al., 2015). Lazarini et al. (2001) demonstrated that deltamethrin did not affect locomotion frequency in rat pups prenatally exposed to deltamethrin at weaning, but decreased general activity after swimming test in male offspring at adult age. In our present study, the fact that there was no difference in swimming speed between the tested groups, implied deltamethrin did not impact swimming capacity in offspring at PND 21. This fact further confirmed the decreased escape latency, swimming distance, and percentage of time spent in the target quadrant in the 9 mg/kg deltamethrin treated group were results of learning and memory deficits induced by prenatal deltamethrin exposure.

It has been reported that the half life for deltamethrin in rat hippocampus was 38.50 h (Anadon et al., 1996), and deltamethrin needs a few days to be completely eliminated from the body (Ruzo et al., 1978). In addition, many studies have provided evidence that deltamethrin exists in mammalian milk for a few days due to its high lipophilicity (Venant et al., 1990; Limon-Miro et al., 2017; Dallegrave et al., 2018). Thus it is plausible that the consumption of deltamethrin via nursing occurred up to weaning and the deltamethrin exposure in this study was likely perinatal even though treatment was prenatal. Furthermore, it is possible that deltamethrin still present in offspring near the time of MWM testing or brain collection. These points present the possibility that cognitive impairment induced by prenatal deltamethrin exposure may be acute effects, not long lasting effects.

The NMDA receptor, a ligand-gated and voltage-dependent glutamate receptor, is composed of an obligatory subunit GluN1 and at least one of the regulatory subunits which include GluN2 (GluN2A, GluN2B, GluN2C, and GluN2D subtypes) and GluN3 (GluN3A and GluN3B subtypes). The different functional attributes of the NMDA receptors are decided by various NMDAR isoforms (Cull-Candy et al., 2001). GluN1, the compulsory subunit of all endogenous NMDARs, is ubiquitously expressed throughout development. GluN2B is predominantly expressed in the neonatal brain but is replaced by or supplemented by GluN2A and GluN2D in the process of development. *N*-methyl-D-aspartate receptors participate in schizophrenia and cognitive disorders such as dementia and Parkinson’s disease, in which GluN1, GluN2A, and GluN2B expression is decreased (Cull-Candy et al., 2001; Zhang et al., 2015). Moreover, NMDARs have been predicted to be involved in the pathological process of pyrethroid-induced neurotoxicity (Chugh et al., 1992; Umeda et al., 2016). Our study found that hippocampal GluN1, GluN2A, and GluN2B protein expression were reduced in offspring whose mothers were treated with deltamethrin at a dose of 9 mg/kg during pregnancy compared with that in the control group (Cull-Candy et al., 2001; Zhang et al., 2015). Therefore, our findings suggested that deltamethrin caused cognitive impairment and that NMDARs were involved in this process. The underlying mechanism might be that deltamethrin, as a type II pyrethroid, caused an extended opening of the Ca^2+^ channel, leading to overactivated NMDA receptors (Morikawa and Furuhama, 2009; Lee et al., 2015) and neural damage, thus inducing cognitive dysfunction (Liu et al., 2010). However, in the groups exposed to relatively lower dose deltamethrin (0.54, 1.35, and 2.7 mg/kg), the expressions of GluN1, GluN2A, and GluN2B were no significantly different from that in the control group. These results are in accordance with previous studies which indicated that pyrethroid-induced neurotoxicity depends on the dose of exposure (Singh et al., 2012).

Brain derived neurotrophic factor, which plays a critical role in synaptic plasticity, neurodevelopment, and neuroprotection through NMDA receptor activation in the hippocampus, binds to tropomyosin-related kinase B (TrkB) receptor, activates intracellular signal transduction pathways, and strengthen synapses and thus improve learning and memory (Li and Liu, 2010). CREB is a molecule located downstream of BDNF and TrkB, and phosphorylation of CREB plays a critical role in the modulation of BDNF in the hippocampus (Amin et al., 2015; Jiang et al., 2017). It has been reported that the neurotrophin BDNF correlates with NMDAR (GluN1, GluN2A, and GluN2B) expression (Caldeira et al., 2007) and that NMDAR activation induces BDNF secretion via Ca^2+^ signals in neurons (Park et al., 2014). Cognitive impairment observed in central nervous system diseases suggests that BDNF may be a potential biomarker candidate as its effect has been implicated in learning and memory (Guo et al., 2015; Zhang et al., 2015). In this study, BDNF, pTrkB/TrkB, and pCREB/CREB expression were reduced in the group exposed to 9 mg/kg deltamethrin, and no significant changes in these protein expressions were detected in the other groups. Consistent with previous studies, our findings showed BDNF expression could be regulated by pyrethroids, which indicated NMDAR/BDNF as possible mediators in the pathogenesis of deltamethrin-induced cognitive dysfunction (Imamura et al., 2000, 2006). The mechanism for this change might be that deltamethrin influences Ca^2+^ homeostasis; excessive Ca^2+^ influx influences NMDAR expression and decreases BDNF secretion, thus reducing pTrkB and downstream pCREB. In contrast to our study, an *in vitro* study found deltamethrin is a potent inducer of BDNF mRNA expression in neurons. This seemingly inconsistent result might be due to differences in the dose and exposure time between our studies (Imamura et al., 2006).

Memantine, a non-competitive, partial NMDAR antagonist, inhibits NMDA glutamate receptors to regulate the glutamatergic system and relieve cognitive and memory deficits in several diseases, such as Alzheimer’s disease, Parkinson’s disease, and ischemia (Olivares et al., 2012; Wang et al., 2016). Previous studies have suggested that memantine prevents excitotoxicity induced by Ca^2+^ overload in neurons via NMDARs and then upregulates BDNF, thereby enhancing cognitive ability (Olivares et al., 2012; Ranju et al., 2015; Liu et al., 2016). The key to the therapeutic action of memantine lies in its uncompetitive binding to NMDARs, through which the low affinity and fast dissociation rate kinetics of memantine maintain the physiological function of the NMDA receptor (Olivares et al., 2012). In our study, GluN1, GluN2A, GluN2B, BDNF, pTrkB/TrkB, and pCREB/CREB, expression level and cognitive abilities were significantly increased in those exposed to both deltamethrin and memantine compared to those in the group exposed to 9 mg/kg deltamethrin alone. These results indicated that memantine could ameliorate the cognitive impairment induced by prenatal deltamethrin exposure in the hippocampus. The possible mechanism might be memantine alleviates excitatory toxicity via providing protection against deltamethrin-induced Ca^2+^ dyshomeostasis, regulating NMDAR expression, promoting BDNF expression, and activating phosphorylation of TrkB and CREB, thus enhancing learning and memory abilities.

## Conclusion

In conclusion, we have found that exposure to a relatively high does of deltamethrin (9 mg/kg) alters cognition in offspring on postnatal day 21 and that this cognitive dysfunction can be ameliorated by memantine treatment. Moreover, NMDAR/BDNF signaling may be associated with the effects of prenatal exposure to deltamethrin on cognitive ability in offspring.

## Ethics Statement

This study was carried out in accordance with the recommendations of Chinese Academy of Sciences. The protocol was approved by the Committee on the Ethics of Animal Experiments of Kunming Medical University.

## Author Contributions

CZ and YL formulated the research questions and designed the study. CZ, QX, and XZ conducted the study. WL and QK carried out the data analysis. CZ wrote the manuscript. TW and XX provided critical review and comments. YL was the supervisor of this research.

## Conflict of Interest Statement

The authors declare that the research was conducted in the absence of any commercial or financial relationships that could be construed as a potential conflict of interest.
