# Corrigendum: Prenatal Deltamethrin Exposure-Induced Cognitive Impairment in Offspring Is Ameliorated by Memantine Through NMDAR/BDNF Signaling in Hippocampus

**DOI:** 10.3389/fnins.2018.00720

**Published:** 2018-10-02

**Authors:** Chao Zhang, Qinghua Xu, Xia Xiao, Weihao Li, Qiang Kang, Xiong Zhang, Tinghua Wang, Yan Li

**Affiliations:** ^1^Department of Women and Child Health, School of Public Health, Kunming Medical University, Kunming, China; ^2^Department of Pediatrics, Weifang Yidu Central Hospital, Shandong, China; ^3^School of Stomatology, Kunming Medical University, Kunming, China; ^4^Department of Hepatobiliary Surgery, The Second Affiliated Hospital of Kunming Medical University, Kunming, China; ^5^Department of Experimental Zoology, Kunming Medical University, Kunming, China

**Keywords:** deltamethrin, *N*-methyl-D-aspartate receptor, cognitive impairment, BDNF, CREB, TrkB

There is an error in the Funding statement. The correct number for National Natural Science Foundation of China is 81673186. The authors apologize for this error and state that this does not change the scientific conclusions of the article in any way.

The original article has been updated.

## Conflict of interest statement

The authors declare that the research was conducted in the absence of any commercial or financial relationships that could be construed as a potential conflict of interest.

